# Incidence and outcomes of refractory immune thrombocytopenic purpura in children: a retrospective study in a single institution

**DOI:** 10.1038/s41598-021-93646-2

**Published:** 2021-07-12

**Authors:** Masataka Ito, Hiroshi Yagasaki, Koji Kanezawa, Katsuyoshi Shimozawa, Maiko Hirai, Ichiro Morioka

**Affiliations:** Pediatrics, Nihon University Itabashi Hospital, 30-1 Ohyaguchi-kamicho, Itabashi-ku, Tokyo, Japan

**Keywords:** Outcomes research, Paediatric research

## Abstract

Treatment of children with refractory immune thrombocytopenic purpura (ITP) is challenging and poorly established. We retrospectively reviewed the clinical data of 87 patients under the age of 16 years who were diagnosed with ITP from April 1998 to March 2017 in our institution. Refractory ITP was defined as a platelet count of < 50 × 10^9^/L at 14 days after receiving intravenous immunoglobulin (IVIG) and prednisolone. We presumed that there was a pathophysiological overlap between refractory ITP and refractory thrombocytopenia (RT): a subtype of refractory cytopenia of childhood (RCC). Immunosuppressive therapies including anti-thymocyte globulin and cyclosporine (CsA) have been adopted for children with RCC in Japan. Thus, from 2009 onwards, we changed the diagnosis from refractory ITP to RT and introduced CsA for refractory ITP/RT. Nine of 42 patients developed refractory ITP in the 1998–2008 group, who received conventional treatments such as IVIG and steroid therapy. Eight of 45 patients developed refractory ITP in the 2009–2017 group, who received CsA with or without IVIG therapy. The response rate at three years after diagnosis was significantly higher in the 2009–2017 group (98%) than in the 1998–2008 group (83%) (*p* = 0.019). In conclusion, our strategy of introducing CsA for refractory ITP/RT contributed to better outcomes.

## Introduction

Immune thrombocytopenic purpura (ITP) is an autoimmune disease characterized by isolated thrombocytopenia and mucocutaneous bleeding. In contrast to adults, ITP in children is self-limiting. However, 20–30% of newly diagnosed ITP patients become refractory to high-dose intravenous immunoglobulin (IVIG) and glucocorticoid therapy^1,2^.

Treatment of children with refractory ITP is challenging and appropriate treatment strategies remain largely unestablished. In 2010, thrombopoietin receptor agonists were authorized as a second-line treatment for adults with chronic ITP in Japan. Forty percent of children with chronic ITP responded to eltrombopag in western countries^3^. Rituximab (RTX) as a second-line treatment was also approved for patients including children with chronic ITP in 2017; however, only 14–26% of children showed a lasting response with a platelet count of > 100 × 10^9^/L^4,5^. The incidence of complete remission in children with chronic ITP who received IVIG, glucocorticoid therapy, or splenectomies was 30–52% at five years^6,7^.

Prior to 2008, patients with refractory ITP underwent administration of glucocorticoid or splenectomy in our institution. However, long term administration of glucocorticoid in children affects bone density as well as growth^8^. Further, splenectomy increases the late risks of venous and arterial thrombosis as well as sepsis from *Streptococcus pneumoniae*^9^. In 2008, refractory cytopenia of childhood (RCC) was introduced as a tentative subtype of pediatric myelodysplastic syndromes (MDS) in the fourth edition of the World Health Organization’s classification of myeloid neoplasms and acute leukemia^10^. We presumed that there was a pathophysiological overlap between refractory ITP and refractory thrombocytopenia (RT), which is a subtype of RCC. Immunosuppressive therapies including anti-thymocyte globulin and cyclosporine (CsA) have been adopted for children with RCC in Japan. From 2009 onwards, we changed the diagnosis from refractory ITP to RT and introduced CsA treatment for such patients instead of glucocorticoid therapy and splenectomy. In this study, we retrospectively analyze the long-term outcomes of 87 children with ITP in our institution and aim to evaluate whether the strategy of introducing CsA for refractory ITP/RT was appropriate.

## Methods

### Data collection

We retrospectively reviewed the clinical data of children who were newly diagnosed with ITP and treated from April 1998 to March 2017 at the Nihon University Itabashi hospital. We excluded patients > 16 years old or with a follow-up duration less than three months from this study. We retrieved the following patient characteristics: age at diagnosis, sex, follow-up time, bleeding manifestations at diagnosis, bone marrow findings, karyotype of bone marrow cells, flow cytometric data on bone marrow, anti-nuclear antibody, platelet count, treatment history, medication history, and outcome at three years after diagnosis. We also classified ITP as newly diagnosed (< 3 months from diagnosis), persistent (3–12 months from diagnosis), and chronic (lasting for > 12 months from diagnosis) according to the criteria of an International Working Group^11^. Informed consents to participate in the study were obtained through providing patients and their guardians with the opportunity to opt-out because this study was a retrospective nature. This procedure was approved by the Institutional Review Board of Nihon University Itabashi hospital and conformed to the revised Declaration of Helsinki, 7th version.

### Response criteria

An international Working Group defined refractory ITP as unresponsive or relapsing after a splenectomy, and requiring treatment to minimize the risk of clinically significant bleeding^11^. In this study, we defined a platelet count of < 50 × 10^9^/L at 14 days after receiving IVIG and prednisolone (PSL) as refractory ITP. Treatment response was defined as either complete response (CR) with a platelet count sustaining > 100 × 10^9^/L or partial response (PR) with a platelet count between 50 and 100 × 10^9^/L.

### Incorporation of CsA

From 2009 onwards, we changed the diagnosis from refractory ITP to RT, and introduced CsA treatment for refractory ITP/RT. We collected the following data: the interval from diagnosis to CsA therapy, the interval from CsA therapy to PR and CR, additional therapies after CsA therapy (IVIG, PSL and their combination), relapse, the duration of CsA therapy, and the outcome at three years after diagnosis.

### Primary outcome and statistical analysis

To evaluate whether the strategy of introducing CsA for refractory ITP/RT contributed to better outcomes, we compared the incidence of CR and PR in ITP at three years after diagnosis between two period groups: 1998–2008 and 2009–2017. Not all patients had been followed up for three years because this study had a retrospective design. If there was no sign of relapse after completion of follow-up, the patient status was defined as CR at three years after diagnosis. Statistical analysis was performed using Jmp, version 14 (SAS Institute Inc., Cary, NC, USA)^12^. Chi-square test and Wilcoxon rank sum test were used to compare patients’ characteristics between the two period groups. Chi-square test was used to compare the incidence of CR and PR between the two period groups. The level of statistical significance was set at *p* < 0.05.

## Results

### Patients’ characteristics

A total of 87 children (50 boys and 37 girls) with ITP were included in this study, of which 42 children were in the 1998–2008 group and 45 children were in the 2009–2017 group. Forty-two children in the 1998–2008 group were classified as follows: 30 patients with newly diagnosed ITP, three patients with persistent ITP, and nine patients with chronic ITP. Further, 45 children in the 2009–2017 group were classified as follows: 36 patients with newly diagnosed ITP, three patients with persistent ITP, and six patients with chronic ITP. The median age at diagnosis was 3.1 years (range, 2 months to 15 years). The median follow-up time was 16 months (range, 3 to 176 months). Bone marrow findings in 86 patients (99%) showed an increased number of mature or immature megakaryocytes, which was consistent with ITP. Bleeding manifestations were present at diagnosis in all patients: skin purpura (99%), nasal bleeding (22%), gingival bleeding (11%), melena (5%), hematuria (2%), intracranial hemorrhage (1%), and genital bleeding (1%). The patients’ characteristics are shown in Table 1 and were comparable between the two period groups. Two patients in the 2009–2017 group had an autoimmune disease; one patient had Sjogren's syndrome and the other systemic lupus erythematosus.

**Table 1:** Patients' characteristics (pre-treatment).

Parameter	1998–2008 group (n = 42)	2009–2017 group (n = 45)	*p*
Median age (range)	3.2 (0.2–15.0)	2.8 (0.2–15.5)	0.96
Boys/Girls	22/20	28/17	0.35
Persistent anti-nuclear antibody (+	5	3	0.39
Autoimmune diseases	0	2	0.166
Baseline platelet level (× 10^9^/L), median (range)	5 (1–19)	7 (1–20)	0.25

### Treatment response to the first-line treatment in all patients with ITP

**Figure 1:**
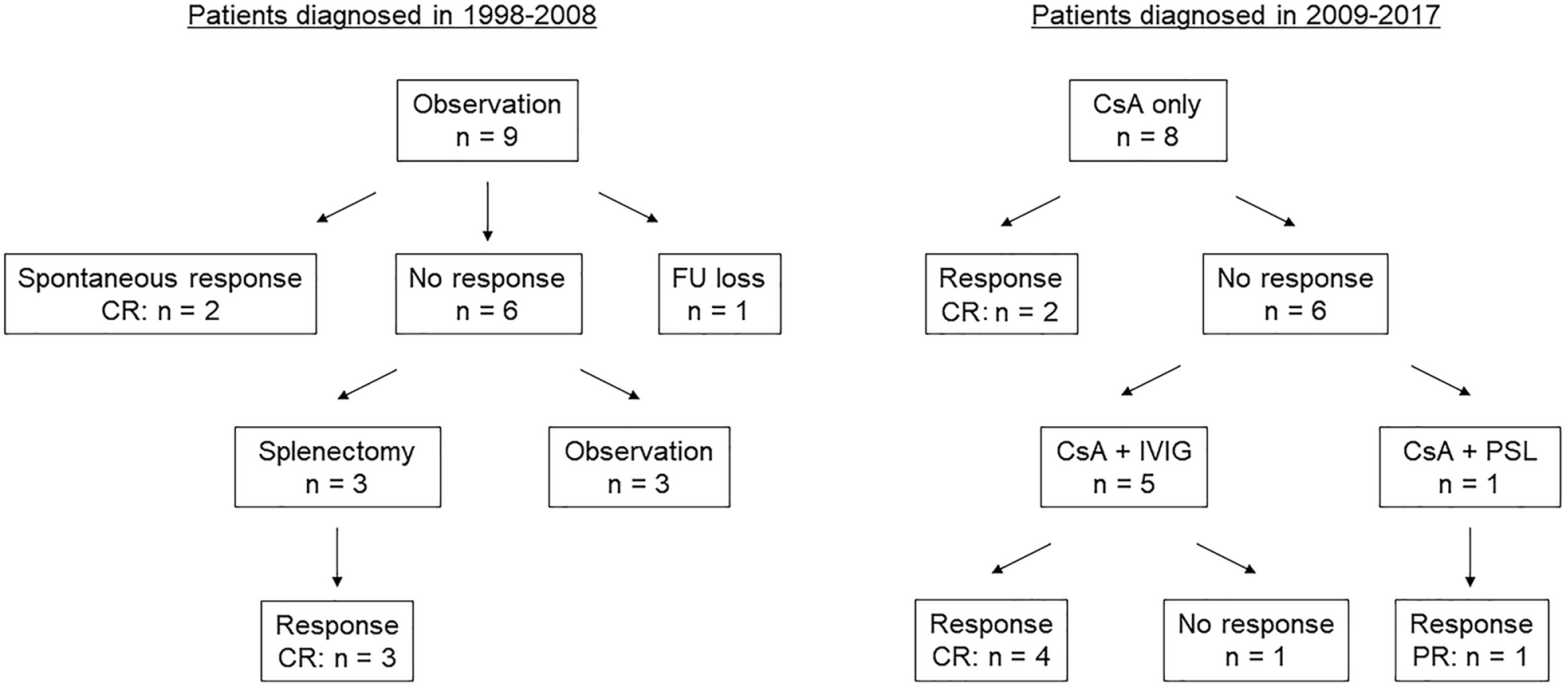
Treatment charts of 17 total patients with refractory immune thrombocytopenic purpura between 1998 and 2017. Nine patients had refractory immune thrombocytopenic purpura in the 1998–2008 group and eight in the 2009–2017 group. In the 1998–2008 group two patients had achieved complete response (CR) without splenectomy. One patient was lost to follow-up (FU). Six patients had a platelet count  < 50 × 10^9^/L at three years after diagnosis, of whom three underwent splenectomy, and CR was achieved at the last follow-up. In the 2009–2017 group two patients achieved CR by cyclosporine (CsA) therapy alone. Six patients did not respond to CsA alone. One patient with Sjogren's syndrome achieved partial response (PR) by adding prednisolone (PSL) to CsA. Four patients had achieved CR by adding intravenous immunoglobulin (IVIG) to CsA. Only one patient failed to respond.

In our institution, the first-line treatments were used for patients presenting with ≤ 20 × 10^9^/L of platelet count and bleeding symptom. All patients with the indication of the first-line treatments received them during the study period. IVIG (1–2 g/kg/day) for 57 patients or PSL (2 mg/kg/day) for 68 patients was administered as the first-line therapy. In the 1998–2008 group, 41 out of 42 patients received PSL as an initial treatment, and 30 patients achieved CR. Out of 11 patients with no response to PSL, 10 patients received IVIG, and two patients achieved CR. The remaining one patient received IVIG as an initial treatment but did not respond to IVIG or addition of PSL. In the 2009–2017 group, 43 out of 45 patients received IVIG as an initial treatment, and 19 patients achieved CR. All 24 patients with no response to IVIG received PSL, and 16 patients achieved CR. The remaining two patients received PSL as an initial treatment and achieved CR by adding IVIG. We found nine patients (21%) in the 1998–2008 group and eight (18%) in the 2009–2017 group with refractory ITP; treatment flow charts of the 17 patients are shown in Fig. 1. Karyotype and flow cytometric analyses showed no abnormal findings in the bone marrow of all patients with refractory ITP when diagnosing ITP.

### Treatment response in the 17 patients with refractory ITP

**Figure 2:**
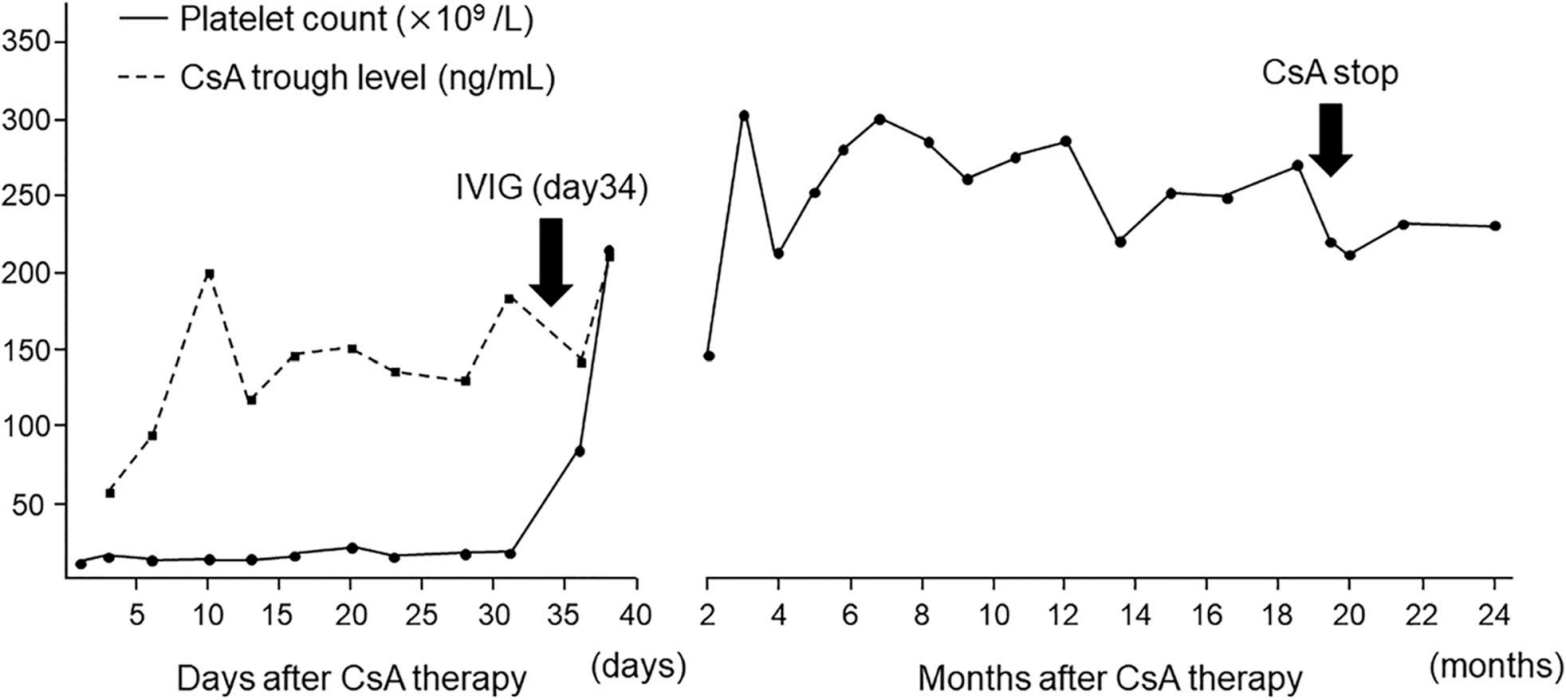
A representative case in which cyclosporine + intravenous immunoglobulin was administered (Patient 3). Treatment with intravenous immunoglobulin (IVIG) and prednisolone did not evoke a response in this patient. The diagnosis of refractory immune thrombocytopenic purpura was changed to refractory thrombocytopenia. No response by cyclosporine (CsA) alone was achieved, and IVIG was added at the CsA trough level 183 ng/mL on day 34. Rapid response was achieved on day 38. Complete response was maintained without additional therapies, and then CsA was discontinued at 19 months.

We changed the diagnosis of refractory ITP for all eight patients in the 2009–2017 group to RT, and initiated treatment with CsA. CsA was administered orally at a dose of 4–6 mg/kg/day and the trough level was adjusted between 100 and 150 ng/mL. Characteristics and outcomes of eight patients who received CsA are presented in Table 2. The median duration from diagnosis to CsA therapy was one month (range, 14 days–10 months). The median administration time of CsA was 19.5 month (range, 5–32 + months). Patient 1 and 2 achieved CR at days 16 and 119 after initiating CsA, respectively. Patient 6 with Sjogren's syndrome achieved PR by addition of PSL. The remaining five patients did not respond to CsA treatment alone, and then received IVIG at the trough level of CsA > 100 ng/mL. With this approach, four patients achieved CR with a median time of 21 days (range, 13–38 days) after CsA administration. A representative case of CsA combined with IVIG therapy in Patient 3 is presented in Fig. 2. Patient 5 responded when receiving second IVIG at the CsA trough level of 147 ng/mL on day 16 after starting CsA therapy. As a result, seven patients (88%) achieved CR and PR owing to the salvage therapy with CsA with or without IVIG. While receiving CsA, three patients (Patient 1, 4 and 7) relapsed with a median time of two months (range, 1–9 months) after CR. Two patients (Patient 1 and 7) obtained CR again by CsA dose adjustment. Patient 4 did not respond to CsA dose adjustment and received IVIG at the trough level of CsA > 100 ng/mL, and then CR was sustained. A total of six patients have maintained CR after CsA discontinuation. Hirsutism was observed in all patients but not severe CsA-associated adverse events. Overall, six out of eight patients (75%) with refractory ITP/RT in the 2009–2017 group sustained CR without any additional medications at three years after diagnosis. Patient 6 with Sjogren's syndrome maintained PR with low-dose CsA (2 mg/kg/day) and PSL (0.2 mg/kg/week) at three years after diagnosis. Only Patient 8 failed to respond and had a platelet count of < 50 × 10^9^/L at three years after diagnosis.

**Table 2:** Characteristics and outcomes of eight patients who received cyclosporine in the 2009–2017 group.

Patient	Age at diagnosis (year)	Sex	Autoimmune disease	Time from diagnosis to CsA therapy	Time to PR after CsA therapy (day)	Time to CR after CsA therapy (day)	Additional IVIG (CsA level (ng/mL))	PSL after CsA therapy	Relapse after CR	Therapy for relapse	Duration of CsA therapy	Status at 3 years after diagnosis
1	1	F	(―)	2 months	10	14	(―)	(―)	1 month	CsA dose adjustment	25 months	CR
2	2	F	(―)	10 months	13	119	(―)	(―)	(―)	(―)	20 months	CR
3	11	M	(―)	24 days	36	38	day 34 (183)	(―)	(―)	(―)	19 months	CR
4	1	F	(―)	1 month	20	20	day 18 (132)	(―)	9 months	CsA dose adjustmentAdditional IVIG	24 months	CR
5	2	M	(―)	14 days	19	22	day 9 (145) day 16 (147)	(―)	(―)	(―)	5 months	CR
6	9	F	Sjogren's syndrome	5 months	15	(―)	(―)	day 106-	(―)	(―)	32 + months	PR
7	1	M	(―)	1 month	13	13	day 10 (100)	(―)	2 months	CsA dose adjustment	5 months	CR
8	6	M	(―)	1 month	(―)	(―)	(―)	(―)	(―)	(―)	14 months	NR

CR, complete response; CsA, cyclosporine; IVIG, intravenous immunoglobulin; NR, no response; PR, partial response; PSL, prednisolone.

In contrast, only two of the nine patients (22%) with refractory ITP achieved CR without splenectomy at three years after diagnosis in the 1998–2008 group. One patient was lost to follow-up. The remaining six patients had a platelet count of < 50 × 10^9^/L at three years after diagnosis, of whom three (50%) underwent splenectomy, and CR was achieved at the last follow up. Characteristics and outcomes of the nine patients with refractory ITP in the 1998–2008 group are presented in Supplementary Table S1 online. As a result, the response rate (CR and PR) at three years after diagnosis was significantly higher in the 2009–2017 group (98%) than in the 1998–2008 group (83%) (*p* = 0.019).

## Discussion

We reported eight pediatric cases with refractory ITP/RT treated with CsA as a salvage therapy with or without IVIG. Surprisingly, seven patients (88%) achieved CR and PR, and six patients (75%) have maintained CR after CsA discontinuation. A Hong Kong group demonstrated that 30 children with persistent or chronic ITP received a salvage therapy with CsA alone^13^. The median administration time of CsA therapy was 9.3 month (range, 0.2–63.9 months), and 11 (37%) and six patients (20%) achieved CR (platelet count > 100 × 10^9^/L) and PR (platelet count between 30 and 100 × 10^9^/L), respectively. Seven out of the 17 patients with CR or PR maintained the response after CsA discontinuation, with the median time of 41.7 months (range, 5.5–116.4 months) from CsA discontinuation. An Italian group demonstrated that 12 adults with refractory ITP received CsA alone, with the median administration time of 14.5 months (range, 3–86 months)^14^. The response (platelet count > 80 × 10^9^/L) was achieved in 10 patients (83%), of whom six patients sustained the response after CsA discontinuation, with the median time of 29.5 months (range, 6–48 months) from CsA discontinuation. An Australian group showed a six-month response rate to a more intensive regimen with dexamethasone + RTX + CsA (platelet count > 30 × 10^9^/L) in 12 out of 20 adults (60%) with persistent or chronic ITP^15^. A more probable response to CsA with or without IVIG for refractory ITP/RT was shown in the current cohort. The dose of CsA was reduced by 10% every month after the platelet count was sustained (> 100 × 10^9^/L) for more than three months. This slowly tapering method was according to that commonly used in patients with aplastic anemia in our country, which might contribute to the high CR rate even after CsA discontinuation.

RCC is characterized by persistent cytopenia with < 2% blasts in the peripheral blood and < 5% blasts in the bone marrow. Moreover, bone marrow films show dysplastic changes in at least two cell lineages, or > 10% within one cell lineage^10^. Interestingly, a common pathogenesis is believed to exist between aplastic anemia and RCC, because immunosuppressive therapies including anti-thymocyte globulin and CsA have been successful for 53–74% of children with RCC^16–18^. RT is a subtype of RCC and is characterized by > 10% morphologic dysplasia limited to the megakaryocytic lineage^10^. However, RT has often been confused with ITP due to the lack of distinguishable dysplasia^19^. Moreover, a case study reported a patient diagnosed with RT using a complementary DNA microarray analysis despite no dysplasia in megakaryocytic lineage^20^. Therefore, we presumed that there was a pathophysiological overlap between refractory ITP and RT.

Autoreactive CD4+ T cells as well as macrophages and B cells are involved in the production of platelet-associated antibodies in adult patients with ITP, which supported our treatment strategy^21^. Our results also indicate that refractory thrombocytopenia is partially mediated by T cells.

In this study, three patients with refractory ITP in the 1998–2008 group underwent splenectomy, and all the patients achieved CR. A retrospective study in pediatric cases showed 85–87% of children with chronic or refractory ITP had a platelet count of > 50 × 10^9^/L after splenectomy^22,23^. Splenectomy has been a conventional treatment for ITP. However, it is not currently preferred because it increases the late risks of venous and arterial thrombosis as well as sepsis from *Streptococcus pneumoniae*^9^. Thus, from 2009 onwards, we avoided splenectomy for refractory ITP owing to these risks. Our study showed that CsA with or without IVIG therapy is feasible and effective in children with refractory ITP to avoid splenectomy.

Our study has some limitations. Firstly, this study followed a retrospective design. Secondly, the study was restricted to a single institution. Moreover, the optimal trough level and treatment duration of CsA remains unestablished.

In conclusion, our strategy of introducing CsA for refractory ITP/RT contributed to better outcomes. Further research is required for optimizing the trough level and duration of CsA.

## Supplementary Information

Supplementary Information.

## Data Availability

The analyzed datasets during the current study are available from the corresponding author on reasonable request.
