# Supplementary material for: Incidence and outcomes of refractory immune thrombocytopenic purpura in children: a retrospective study in a single institution
Source: Sci Rep. 2021 Jul 12;11:14263. doi: 10.1038/s41598-021-93646-2 (PMC8275793; doi:10.1038/s41598-021-93646-2)
Supplement: Supplementary file 1 — Supplementary Information. [file 41598_2021_93646_MOESM1_ESM.pdf]

Incidence and outcomes of refractory immune thrombocytopenic purpura in children: a retrospective study in a single institution

Masataka Ito, *Hiroshi Yagasaki, Koji Kanezawa, Katsuyoshi Shimozawa, Maiko Hirai, and Ichiro Morioka

Supplementary Table S1. Characteristics and outcomes of the nine patients with refractory immune thrombocytopenic purpura in the 1998-2008 group

Patient	Age at diagnosis (year)	Sex	Autoimmune disease	ANA	Status at 3 years after diagnosis	Splenectomy	Age at Splenectomy (year)	CR after Splenectomy
1	13	M	(-)	(+)	NR	(+)	20	(+)
2	0	F	(-)	(-)	NR	(-)		
3	3	F	(-)	(-)	NR	(+)	8	(+)
4	11	F	(-)	(+)	NR	(-)		
5	4	F	(-)	(-)	NR	(-)		
6	2	F	(-)	(-)	CR	(-)		
7	9	F	(-)	(-)	NR	(+)	13	(+)
8	0	F	(-)	(-)	CR	(-)		
9	2	F	(-)	(-)	Follow-up loss	(-)		

ANA, antinuclear antibodies; CR, complete response; NR, no response; PR, partial response
